# Exploring internal features of 16S rRNA gene for identification of clinically relevant species of the genus *Streptococcus*

**DOI:** 10.1186/1476-0711-10-28

**Published:** 2011-06-25

**Authors:** Devi Lal, Mansi Verma, Rup Lal

**Affiliations:** 1 Molecular Biology Laboratory, Department of Zoology, University of Delhi, Delhi-110007, India

**Keywords:** phylogenetic framework, signature sequences, genetic heterogeneity

## Abstract

**Background:**

*Streptococcus *is an economically important genus as a number of species belonging to this genus are human and animal pathogens. The genus has been divided into different groups based on 16S rRNA gene sequence similarity. The variability observed among the members of these groups is low and it is difficult to distinguish them. The present study was taken up to explore 16S rRNA gene sequence to develop methods that can be used for preliminary identification and can supplement the existing methods for identification of clinically-relevant isolates of the genus *Streptococcus*.

**Methods:**

16S rRNA gene sequences belonging to the isolates of *S. dysgalactiae, S. equi*, *S. pyogenes*, *S. agalactiae*, *S. bovis*, *S. gallolyticus*, *S. mutans*, *S. sobrinus, S. mitis*, *S. pneumoniae*, *S. thermophilus *and *S. anginosus *were analyzed with the purpose to define genetic variability within each species to generate a phylogenetic framework, to identify species-specific signatures and *in-silico *restriction enzyme analysis.

**Results:**

The framework based analysis was used to segregate *Streptococcus *spp. previously identified upto genus level. This segregation was validated using species-specific signatures and *in-silico *restriction enzyme analysis. 43 uncharacterized *Streptococcus *spp. could be identified using this approach.

**Conclusions:**

The markers generated exploring 16S rRNA gene sequences provided useful tool that can be further used for identification of different species of the genus *Streptococcus*.

## Background

The genus *Streptococcus *consists of spherical Gram positive bacteria belonging to the class *Bacilli *and the order *Lactobacillales *[1]. The group is large and comprises of numerous clinically significant species which are responsible for wide variety of infections in human and animals. *Streptococcus *of different groups are known to cause human diseases, some species being highly virulent and responsible for major diseases. Species like *S. pyogenes*, *S. agalactiae *and *S. pneumoniae *are important as they cause serious acute infections in man, but several other species are also involved in a number of diseases like infective endocarditis, abscesses and other pathological conditions [2]. Various species of *Streptococcus *are known to be associated with infections of cattles, pigs, horses, sheeps, birds, aquatic mammals and fishes [3]. The genus has undergone considerable taxonomic revisions and has been divided into different groups (pyogenic, anginosus, mitis, mutans, salivarius, bovis) based on 16S rRNA gene sequence similarity [4].

Since many species belonging to the genus *Streptococcus *are associated with various pathological conditions, different protocols have been used for their identification. Still precise identification of these species is laborious. Clinical laboratories use serological grouping by Lancefield, haemolytic reactions and phenotypic tests for identification of various *Streptococcus *isolates. However, these Lancefield groups are not species-specific [5,6] and haemolytic activity differs within species and depends on incubation procedures. Strains within a given species may differ for a common trait [7,8] and even the same strain may exhibit biochemical variability [9,10].

Various alternatives have been employed for identification of *Streptococcus *isolates. These include DNA hybridization [11-14]; rDNA restriction analysis [15-17]; use of 16S-23S rRNA interspacer region [18-21], D-alanyl-D-alanine ligase gene (*ddl *gene) [22]; autolysin gene (*lytA*) [23]; dextranase (*dex*) [24]; heat shock protein (*groESL*) [25,26]; RNA subunit of endoribonuclease P (*rnpB*) [27]; the elongation factor Tu (*tuf*) [28]; gyrase A (*gyrA*) and topoisomerase subunit C (*parC*) [29]; sequence analysis of small subunit rRNA [30]; manganese-dependent superoxide dismutase gene (*sodA*) [31,32]; recombination and repair protein (*recN*) [33]; tDNA PCR fragment length polymorphism [34,35] and use of multilocus sequencing typing loci [36,37].

16S rRNA gene sequencing has proved to be one of the most powerful tools for the classification of microorganisms [38] and has been used for identification of clinically relevant microbes [39,40]. Therefore, molecular tools based on 16S rRNA gene can be developed and used for identification. However it is also true that the correct identification of bacterial species may not be based on the nucleotide sequence of a single gene. Multilocus Sequence Analysis (MLSA) of several house-keeping genes has to be performed. But from practical standpoint there is need for a simplified approach for preliminary identification of a species, particularly under the conditions if the amount of isolated DNA is not enough for MLSA or it does not react with a complete set of typing primers. The current work considers the possibility to use 16S rRNA sequences for this purpose and is useful for practical applications.Thus the present study aims to explore internal features of 16S rRNA gene for preliminary identification of a species that can supplement the existing methods for identification. These methods include construction of phylogenetic framework, identification of species-specific signatures and restriction enzyme analysis.

## Methods

### Sequence data

16S rRNA gene sequences belonging to the genus *Streptococcus *from RDP database http://rdp.cme.msu.edu/[41] were analysed in the present study. These included the sequences with relatively high number of identified organisms (86 sequences belonging to isolates of *S. dysgalactiae*, 61 to *S. equi*, 61 to *S. pyogenes*, 29 to *S.agalactiae*, 31 *S. bovis-equinus *(*S. bovis *and *S. equinus *are considered to be a single species [42]), 76 to *S. gallolyticus*, 102 to *S. mutans*, 23 to *S.sobrinus*, 28 to *S. mitis*, 41 to *S. pneumoniae*, 73 to *S. thermophilus*, 32 to *S. anginosus*) and 63 sequences of uncharacterized species identified only upto genus level. The sequences belonging to twelve sets of *Streptococcus *species occurring with higher frequency were used as the master species set for generating a phylogenetic framework, species-specific signatures and restriction enzyme analysis.

### Phylogenetic Analyses

For phylogenetic analyses, the sequences were aligned using multiple alignment program CLUSTAL_X [43]. Evolutionary distances between all the sequences were calculated with DNADIST of the PHYLIP 3.6 package [44]. The program NEIGHBOR was used to draw neighbor joining [45] tree with Jukes and Cantor correction [46]. Statistical testing of the trees was done using SEQBOOT by resampling the dataset 1000 times. The trees were viewed through TreeView Version 1.6.6 [47]. For each of these 12 *Streptococcus *species data sets, sequences that formed a single cluster were aligned and a consensus was obtained by using JALVIEW sequence editor [48]. The sequence close to consensus from each group was chosen as a representative for that particular group. Based on this, a reference set of 63 sequences was selected to define the range of genetic variability present in each of the *Streptococcus *species.

### Specific Signatures

Signatures were identified in each of the species data set using online MEME program [49]. Sequences of 12 *Streptococcus *species data sets were submitted groupwise in MEME program Version 4.6.1 http://meme.sdsc.edu/meme4_6_1/cgi-bin/meme.cgi. In order to obtain maximum number of motifs the default setting was modified from 3 motifs to 20 motifs. The default value of motif widths was also modified and re-set between 25 and 50. Each of the 20 signatures was checked for its frequency of occurrence among a particular *Streptococcus *sp. The signatures which did not appear in other *Streptococcus *spp. were considered as unique. BLAST search against NCBI database http://www.ncbi.nlm.nih.gov/ was carried out for these signatures to check their uniqueness.

### Restriction enzyme analysis

Eleven Type II Restriction enzymes (Table 1) were considered for these analyses. Restriction Mapper Version 3 http://restrictionmapper.org/ was used to obtain the restriction pattern of the 12 *Streptococcus *species data sets employed for construction of phylogenetic framework. These restriction patterns were analyzed and a consensus pattern was determined for each species.

**Table 1:** Restriction enzymes used in the present study

S.No.	Restriction enzyme	Cut site
1.	***Alu*I**	AG**^▼^**CT

2.	***Bam*HI**	G**^▼^**GATCC

3.	***Bfa*I**	C**^▼^**TAG

4.	***Eco*RI**	G**^▼^**AATTC

5.	***Hae*III**	GG**^▼^**CC

6.	***Hha*I**	GCG**^▼^**C

7.	***Hind*III**	A**^▼^**AGCTT

8.	***Msp*I**	C**^▼^**CGG

9.	***Rsa*I**	GT**^▼^**AC

10.	***Sau*3AI**	**^▼^**GATC

11.	***Sma*I**	CCC**^▼^**GGG

### Cluster analysis for restriction profile

For cluster analyses MVSP (Multi Variate Statistical Package, Kovach Computing Services) version 3.13p was used. Dendrograms were constructed using the restriction patterns generated by different restriction enzymes for 12 framework species. The dendrograms show the utility of these enzymes in distinguishing different strains.

## Results

In the present study, 16S rRNA gene sequences belonging to 12 different species from the genus *Streptococcus *were analyzed with the aim to construct phylogenetic framework, identification of species-specific signatures and restriction enzyme analysis.

### Phylogenetic framework

**Figure 1:**
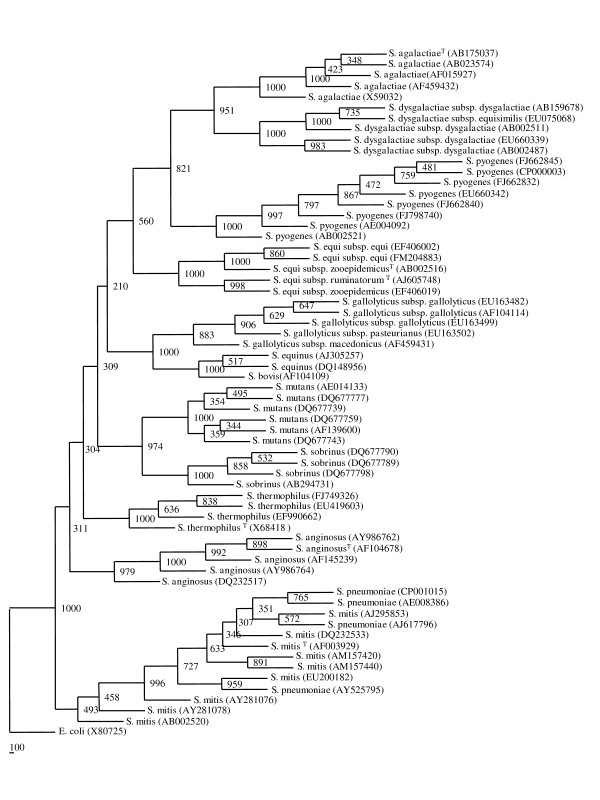
**Phylogenetic tree based on 63 representative 16S rRNA gene sequences from 12 *Streptococcus *species**. The tree was constructed by neighbour-joining method with Jukes and Cantor correction. The numbers at node represent bootstrap values (based on 1000 resampling). The accession numbers are shown in parenthesis.

Phylogenetic tree (Additional file 1: Fig. S1) based on 61 sequences of *S. pyogenes *revealed 8 clusters. 8 sequences representing these 8 clusters were chosen. These sequences could represent genetic heterogeneity present within this species. Similarly, sequences from other *Streptococcus *spp. were analysed for genetic heterogeneity present within them (Additional file 2, 3, 4, 5, 6, 7, 8, 9, 10, 11, 12: Fig. S2-S12). Different representative sequences were choosen from each species that could provide information regarding the range of genetic variability present within each species (Table 2). 63 such representative sequences were selected for framework construction (Figure 1). Strains of all the species were clearly segregated except for *S. pneumoniae *and *S. mitis *suggesting a high level of similarity between strains of these two species, making their identification difficult solely on the basis of 16S rRNA gene.

**Table 2:** Sequences used for generating phylogenetic framework.

Species	No. of sequences used	No. of clusters obtained	No. of representative sequences	Accession number of representative sequences
***S. dysgalactiae***	86	5	5	AB002487, EU660339, AB002511, AB159678, EU075068

***S. equi***	61	5	5	EF406002, FM204883, EF406019, AB002516^T^, AJ605748^T^

***S. pyogenes***	61	8	8	FJ798740, FJ662832, FJ662840, AB002521, AE004092, FJ662845, CP000003, EU660342

***S. agalactiae***	29	4	5	AF459432, AB023574, AF015927, AB175037^T^, X59032

***S. bovis-equinus***	31	3	3	AF104109, AJ305257, DQ148956

***S. gallolyticus***	76	5	5	AF104114, EU163502, EU163482, EU163499, AF459431

***S. mutans***	102	6	6	DQ677759, DQ677739, DQ677777, AE014133, AF139600, DQ677743,

***S. sobrinus***	23	4	4	AB294731, DQ677789, DQ677790, DQ677798

***S. mitis***	28	7	9	AF003929^T^, AJ295853, EU200182, AM157440, DQ232533, AM157420, AY281076, AB002520, AY281078

***S. pneumoniae***	41	3	4	AE008386, CP001015, AJ617796, AY525795

***S. thermophilus***	73	3	4	EF990662, X68418^T^, EU419603, FJ749326

***S. anginosus***	32	5	5	AY986762, AF145239, AF104678^T^, AY986764, DQ232517

**Total**	**643**	**59**	**63**	

Type strains are denoted by "T" as superscript.

**Figure 2:**
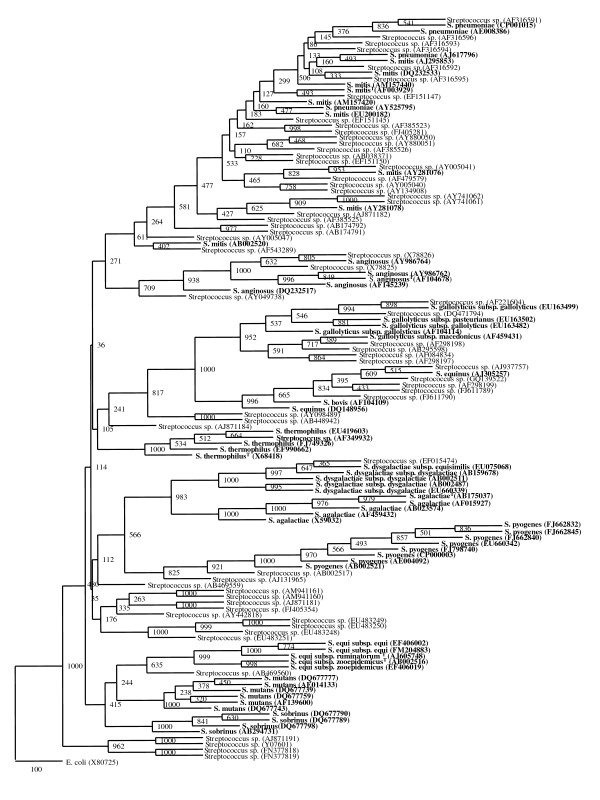
**Phylogenetic tree of 63 framework sequences (bold values) and uncharacterized *Streptococcus *spp**. The tree was constructed by neighbour-joining method with Jukes and Cantor correction. The numbers at node represent bootstrap values (based on 1000 resampling). The accession numbers are shown in parenthesis.

The framework generated was then used to check if uncharacterized *Streptococcus *spp. can be classified among the framework species (Figure 2). Out of 63 sequences previously identified upto genus level, 43 were found to segregate with 7 *Streptococcus *framework species, supported by high bootstrap values. Among these 43 sequences, 3 segregated with *S. anginosus*, 21 with *S. mitis*, 6 with *S. pneumoniae *and *S. gallolyticus *each, 5 with *S. bovis-equinus*, 1 with *S. dysgalactiae *and *S. thermophilus *each (Figure 2). No strains could be segregated with *S. mutans, S. pyogenes, S. equi, S. agalactiae *and *S. sobrinus*. The framework based segregation was further validated by checking for the presence of species-specific signatures and restriction analysis.

### Signature sequences

Out of 20 signatures identified for each of the 12 *Streptococcus *framework species, only 1-5 unique signatures were found (Table 3) in framework species. The unique signatures were found in: *S. mutans*: 5; *S. dysgalactiae*: 3; *S. equi*, S*. sobrinus *and *S. thermophilus*: 2 each; *S. gallolyticus*, *S. agalactiae, S. pyogenes, S. bovis-equinus, S. anginosus *and *S. pneumoniae*: 1 each. These signatures were found to occur with high frequency. Moreover, these signatures were also found to be highly conserved across a particular species showing 98-100% sequence identity but were found to be fragmented in other species. No unique signature could be identified for *S. mitis*. But *S. mitis *can still be distinguished from *S. pneumoniae *using the signature found unique to *S. pneumoniae*. In *S. mitis *the signature was found to be substituted at specific positions and thus can distinguish these two species (Table 3). The signature found for *S. bovis-equinus *was effective in distinguishing it from very closely related species like *S. lutetiensis *and *S. gallolyticus*. These signatures were further used to validate the segregation of 43 sequences among 7 different framework species. All 43 sequences were found to contain the signature unique to the particular species thus validating the affiliation of these sequences to a particular species.

**Table 3:** Unique signature sequences identified for 12 *Streptococcus *framework spp.

*Streptococcus sp*.	Unique Signature (Nucleotides)	Frequency of occurance
***S. dysgalactiae***	AATACA(G)TGCAAGTAGAACGCTGAGGACTGGTGCTTGCACCGGTCCAAGGA (52-101)	77/86
	**TGCATCACTATGAGATGGACCTGCG**TTGTATTAGCTAGTTGGTGAGGTAA (223-272)	86/86
	CATTTAAAAGGTGCAAT**TGCATCACTATGAGATGGACCTGCG **(207-246)	86/86

***S. equi***	AAGAAGGTTTTCGGATCGTAAAGCTCTGTTGTTAGAGAAGAACAGTGATG (420-469)	61/61
	AAAGTCCATCATGTGACGGTAACTAACCAGAAAGGGACGGCTAACTACGT (476-525)	61/61

***S. pyogenes***	AAACGATAGCTAATACCGCATAAGAGAGACTAACGCATGTTAGTAATTTA (172-222)	56/61

***S.agalactiae***	GCAGTGGCTTAACCATTGTACGCTTTGGAAACTGGAGGACTTGAGTGC (613-660)	29/29

***S. bovis***	GCNTTTAACNCATGTTAGPuNGCTTGAAPuGPuAGCAA (178-212)	31/31

***S. gallolyticus***	TCTTGACATCCCGATGCTATTTCTAGAGATAGAAAGTTTCTTCGGAACAT (991-1040)	76/76

***S. mutans***	AGTAAAAGGCTATGGCTCAACCATAGTGTGCTCTGGAAACTGTCTGACTT (611-660)	102/102
	ACCTGGGCTACACACGTGCTACAATGGTCGGTACAACGAGTTGCGAGCCG (1222-1271)	102/102
	ATGATAATTGATTGAAAGATGCAAGCGCATC**ACTAGTAGATGGACCTGCG **(197-246)	102/102
	**ACTAGTAGATGGACCTGCG**TTGTATTAGCTAGTTGGTAAGGTAAGAGCTT (228-277)	102/102
	AACACACTGTGCTTGCACACCGTGTTTTCTTGAGTCGCGAACGGGTGAGT (70-119)	102/102

***S.sobrinus***	TCACACCACGAGAGTTTGTAACACCCAAAGTCGGTGAGGTAACCATTTAT (1417-1466)	23//23
	AAGTGGAACGCATTGGTAACACCGGACTTGCTCCAGTGTTACTAATGAGT (54-103)	23/23

***S. mitis***	TPuPyGCATGACPyANPyTNNNTTPuAAAGGTGCANTTGCAPyCACTANNAGATGGA (228-277)	28/28

***S. pneumoniae***	TGTTGCATGACATTTPuCTTAAAAGGTGCANNTGCATCACTACCAGATGGA (179-228)	41/41

***S. Thermophilus***	ACAATGGTTGGTACAACGAGTTGCGAGTCGGTGACGGCGAGCTAATCTCT (1246-1295)	73/73
	AAGATGGACCTGCGTTGTATTAGCTAGTAGGTGAGGTAATGGCTCACCTA (234-283)	73/73

***S. anginosus***	ATTTATTGGGCGTAAAGCGAGCGCAGGCGGTTAGAAAAGTCTGAAGT (581-530)	32/32

The signatures were found to 98-100% identical and conserved in a particular species. Py denotes pyrimidine, Pu denoted purine and N denotes any nucleotide. Overlapping signatures are shown in bold.The number in brackets indicate the corresponding position of the signature in 16S rRNA gene.

### Restriction enzyme analysis

In-silico restriction enzyme analysis using eleven type II enzymes revealed different patterns. Restriction sites for *Alu*I, *Bfa*I, *Hae*III, *Msp*I, *Rsa*I and *Sau*3AI occurred with frequency of 3-10 resulting in 4-11 fragments. The sites for enzymes *Eco*RI, *Sma*I and *Hha*I were found in majority of sequences studied but they were found to be less frequent cutters producing single, single and double cuts respectively. These enzymes thus are less informative and serve no purpose. Inspite of low frequency, *Bam*HI and *Hind*III can still be used for distinguishing different *Streptococcus *spp. *Bam*HI produced single but unique cut in *S. thermophilus *and can be used to distinguish *S. thermophilus *isolates. *Hind*III produces single but unique cut in *S. sobrinus *and can be used to distinguish *S. sobrinus *isolates. As can be seen from the dendrograms (Additional file 13, 14, 15, 16, 17, 18: Fig. S13-S18) different restriction enzymes can be used for identification and distinguishing different isolates. While *Alu*I was found to distinguish majority of *Streptococcus *framework species, *Msp*I produced maximum numbers of cuts and thus proved informative for such analysis. Closely related species like *S. dysgalactiae *and *S. agalactiae *can be distinguished using *Bfa*I, *Msp*I and *Hae*III. Similarly, *S. gallolyticus *and *S. bovis *can be distinguished using *Bfa*I and *Hae*III. Therefore a combination of *Alu*I, *Bfa*I, *Msp*I or *Hae*III can be used for distinguishing closely related organisms. The sequences segregated with framework species were further validated using *in-silico*restriction enzyme analysis. The identified sequences showed unique restriction enzyme pattern close to the nearby framework species (Table 4) again validating the framework based segregation. Thus combining the information from framework, signature sequences and restriction enzyme analysis it was possible to identify 43 sequences (out of 63) upto species level which were previously designated as *Streptococcus *sp. (Table 4).

**Table 4:** *Streptococcus *spp. identified upto species level:

S. No.	Acccession no.	Close framework species	Presence of species-specific signatures	Unique RE pattern
**1**	EF015474	*S. dysgalactiae*	Y	*S. dysgalactiae *(*Msp*I)

**2**	AF221604			
	DQ471794			
	AF298198	*S. gallolyticus*	Y	*S. gallolyticus *(*Bfa*I)
	AB295598			
	AF084834			
	AF298197			

**3**	AF349932	*S. thermophilus*	Y	*S. thermophilus *(*Alu*I, *Hae*III *Sau*3AI,*Bam*HI)

**4**	X78826			
	X78825	*S. anginosus*	Y	*S. anginosus *(*Alu*I, *Bfa*I, *Hae*III)
	AY049738			

**5**	AF316591			
	AF316596			
	AF316593	*S. pneumonaie*	Y	*S. pneumonaie *(*Alu*I, *Msp*I)
	AF316594			
	AF316595			
	EF151147			

**6**	AF316592			
	EF151145			
	AF385523			
	FJ405281			
	AY880050			
	AY880051			
	AF385526			
	AB038371			
	EF151150	*S. mitis*	Y	*S. mitis *(*Alu*I, *Msp*I, *Sau*3AI)
	AY005041			
	AF479579			
	AY005040			
	AY134908			
	AY741062			
	AY741061			
	AJ871182			
	AF385525			
	AB174792			
	AB174791			
	AY005047			
	AF543289			

**7**	AJ937757	*S. bovis-equinus*	Y	*S. bovis-equinus *(*Bfa*I)
	GQ139522			
	AF298199			
	FJ611789			
	FJ611790			

## Discussion

*Streptococcus *is a clinically important genus as a number of species belonging to this genus are human and animal pathogens. This genus has undergone considerable taxonomic revisions and has been divided into different groups based on 16S rRNA gene sequence similarity.

The present study aims to explore internal features of 16S rRNA gene sequences of different *Streptococcus *spp. to develop methods for their identification. A phylogenetic framework was constructed using different representative sequences followed by identification of signature sequences and restriction enzymes analysis. The framework based analysis suggests a high level of genetic heterogeneity present within different *Streptococcus *spp. Signature sequences specific for each *Streptococcus *framework sp. were identified. These signature motifs would be simple to use as a supplement to the automated identification process. Restriction analysis has proved to be an important tool to identify newly isolated strains [50-52] and can be exploited for describing new species [53]. Multiple restriction enzyme usage is recommended for better resolution. Although it has been documented that closely related species cannot be distinguished solely on the basis of 16S rRNA gene, but exploring the internal features of this gene can be of definite use. Therefore researchers are now looking to explore the unique features of 16S rRNA gene that have not been explored yet [54,55]. As already described the genus *Streptococcus *has been divided into different groups based on 16S rRNA gene similarity [4]. The framework species used for these analyses belong to these different groups.

Four framework species- *S. pyogenes, S. agalactiae, S. equi *and *S. dysgalactiae *belong to pyogenic group which is the largest group of the genus *Streptococcus*. *S. pyogenes, S. agalactiae, S. equi *and *S. dysgalactiae *can be distinguished on the basis of specific signatures (Table 3) as well as using different restriction enzymes (Additional file 13, 14, 15, 16, 17, 18: Figures S13-S18).

Two framework species, *S. pneumoniae *and *S. mitis *belong to mitis group. Identification of members of mitis group, particularly *S. pneumoniae *is problematic. Identification of *S. pneumoniae *isolates is usually done using serological [56,57] and molecular techniques [58-60]. *S. pneumoniae *isolates can be easily identified using the signature sequence as given in Table 3. We could easily distinguish *S. pneumoniae *isolates from *S. mitis *using this signature sequence. Other members of this group (*S.mitis *and *S. oralis*) that are almost indistinguishable on the basis of complete 16S rRNA gene sequence can be differentiated using different restriction enzymes. *S. mitis *can be distinguished from other two species of this group by using enzyme *Sau*3AI. *S. pneumoniae*, *S.mitis *and *S. oralis *can be distinguished from each other by exploiting *Alu*I and *Msp*I (data not shown for *S. oralis*).

Framework species *S. anginosus *belongs to anginosus group. This group consists of only 3 species (*S. anginosus*, *S. intermedius *and *S. constellatus*). Members of anginosus group are also difficult to identify and distinguish. An identification scheme for differentiation of these 3 strains was proposed by Whiley *et al. *[61] and Whiley and Beighton [62]. Commercial identification systems [63,64] and molecular methods have been used for identifying and distinguishing these three species [65,14]. *S. anginosus *can be easily identified using the signature sequence (Table 3) and use of restriction enzymes. Restriction enzymes *Alu*I, *BfaI, Rsa*I and *Hae*III can be used for distinguishing members of anginosus group efficiently (data not shown for *S. intermedius *and *S. constellatus*).

Framework species, *S. thermophilus *belongs to salivarius group which is closely related to bovis group [66] and consists of only 3 species (*S. salivarius, S. vestibularis, S. thermophilus*). *S. thermophilus *can be identified by using unique signature sequence (Table 3).

Two framework species, *S. bovis *and *S. gallolyticus *belong to bovis group. Members of bovis group, *S. bovis *and *S. gallolyticus *are difficult to identify. Isolates of these two species can be distinguished using the signature specific for *S. gallolyticus *and *S. bovis*. Moreover, the use of restriction enzymes *Alu*I, *Bfa*I and *Hae*III can be instrumental in distinguishing them. The signature found for *S. bovis *was found to be efficient in distinguishing *S. bovis *from closely related species-*S. lutetiensis*. These two species are difficult to distinguish solely on the basis of 16S rRNA gene.

Framework species *S. mutans *and *S. sobrinus *belong to mutans group. These two species are also difficult to distinguish. Beighton *et al. *(1991) [8] provided a scheme for identification of *S. mutans *and *S. sobrinus *strains. In the present investigations these two can be easily distinguished using species-specific signature and use of restriction enzymes- *Alu*I, *Bfa*I, *Hae*III, *Msp*I, *Rsa*I and *Sau*3AI.

## Conclusions

The species that are difficult to distinguish solely on the basis of 16S rRNA gene sequence can be identified using inner secrets of 16S rRNA gene, the signatures. These signatures can be exploited for quick identification. The aim of phylogenetic framework construction is to define a range of genetic variability within the species and later exploiting this variability for identification of different isolates. Similarly, use of restriction enzymes help in generating markers that can distinguish closely related species. Present study reveals that the framework, use of specific signatures in 16S rRNA gene and pattern generated by different restriction enzymes can be exploited for identification of isolates belonging to the genus *Streptococcus*. The markers generated in the present study are based on 16S rRNA gene sequence which is conserved and neither subjected to changes due to culture conditions nor exhibit biochemical variability. Thus the scheme proposed can be applied to any isolate. The approach is cost effective and rapid way for identification of various isolates and thus can be used to differentiate isolates that are difficult to distinguish due to very close traits and biochemical features. Additionally the approach is simple for preliminary identification of a species and can supplement existing automated identification processes. But we should keep in mind that this is a simplified procedure and thus it is also important to know the limitations of such simplified approach.

## Competing interests

The authors declare that they have no competing interests.

## Authors' contributions

DL, MV and RL conceived and designed the experiments. DL and MV performed the experiments. DL, MV and RL analyzed the data. DL, MV and RL wrote the manuscript. All authors read and approved the final manuscript.

## Supplementary Material

Additional file 1 **Phylogenetic tree based on 61, 16S rRNA gene sequences of *Streptococcus pyogenes***. The tree was constructed by neighbour-joining method with Jukes and Cantor correction. The numbers at node represent bootstrap values (based on 1000 resampling). The accession numbers are shown in parenthesis. Bold sequences indicate those which are used for final framework construction. Click here for file

Additional file 2 **Phylogenetic tree based on 86, 16S rRNA gene sequences of *Streptococcus dysgalactiae***. The tree was constructed by neighbour-joining method with Jukes and Cantor correction. The numbers at node represent bootstrap values (based on 1000 resampling). The accession numbers are shown in parenthesis. Bold sequences indicate those which are used for final framework construction. Click here for file

Additional file 3 **Phylogenetic tree based on 29, 16S rRNA gene sequences of *Streptococcus agalactiae***. The tree was constructed by neighbour-joining method with Jukes and Cantor correction. The numbers at node represent bootstrap values (based on 1000 resampling). The accession numbers are shown in parenthesis. Bold sequences indicate those which are used for final framework construction. Click here for file

Additional file 4 **Phylogenetic tree based on 61, 16S rRNA gene sequences of *Streptococcus equi***. The tree was constructed by neighbour-joining method with Jukes and Cantor correction. The numbers at node represent bootstrap values (based on 1000 resampling). The accession numbers are shown in parenthesis. Bold sequences indicate those which are used for final framework construction. Click here for file

Additional file 5 **Phylogenetic tree based on 31, 16S rRNA gene sequences of *Streptococcus bovis-equinus***. The tree was constructed by neighbour-joining method with Jukes and Cantor correction. The numbers at node represent bootstrap values (based on 1000 resampling). The accession numbers are shown in parenthesis. Bold sequences indicate those which are used for final framework construction. Click here for file

Additional file 6 **Phylogenetic tree based on 76, 16S rRNA gene sequences of *Streptococcus gallolyticus***. The tree was constructed by neighbour-joining method with Jukes and Cantor correction. The numbers at node represent bootstrap values (based on 1000 resampling). The accession numbers are shown in parenthesis. Bold sequences indicate those which are used for final framework construction. Click here for file

Additional file 7 **Phylogenetic tree based on 102, 16S rRNA gene sequences of *Streptococcus mutans***. The tree was constructed by neighbour-joining method with Jukes and Cantor correction. The numbers at node represent bootstrap values (based on 1000 resampling). The accession numbers are shown in parenthesis. Bold sequences indicate those which are used for final framework construction. Click here for file

Additional file 8 **Phylogenetic tree based on 23, 16S rRNA gene sequences of *Streptococcus sobrinus***. The tree was constructed by neighbour-joining method with Jukes and Cantor correction. The numbers at node represent bootstrap values (based on 1000 resampling). The accession numbers are shown in parenthesis. Bold sequences indicate those which are used for final framework construction. Click here for file

Additional file 9 **Phylogenetic tree based on 28, 16S rRNA gene sequences of *Streptococcus mitis***. The tree was constructed by neighbour-joining method with Jukes and Cantor correction. The numbers at node represent bootstrap values (based on 1000 resampling). The accession numbers are shown in parenthesis. Bold sequences indicate those which are used for final framework construction. Click here for file

Additional file 10 **Phylogenetic tree based on 41, 16S rRNA gene sequences of *Streptococcus pneumoniae***. The tree was constructed by neighbour-joining method with Jukes and Cantor correction. The numbers at node represent bootstrap values (based on 1000 resampling). The accession numbers are shown in parenthesis. Bold sequences indicate those which are used for final framework construction. Click here for file

Additional file 11 **Phylogenetic tree based on 32, 16S rRNA gene sequences of *Streptococcus anginosus***. The tree was constructed by neighbour-joining method with Jukes and Cantor correction. The numbers at node represent bootstrap values (based on 1000 resampling). The accession numbers are shown in parenthesis. Bold sequences indicate those which are used for final framework construction. Click here for file

Additional file 12 **Phylogenetic tree based on 73, 16S rRNA gene sequences of *Streptococcus thermophilus***. The tree was constructed by neighbour-joining method with Jukes and Cantor correction. The numbers at node represent bootstrap values (based on 1000 resampling). The accession numbers are shown in parenthesis. Bold sequences indicate those which are used for final framework construction. Click here for file

Additional file 13 **Dendrogram based on restriction digestion of 12 *Streptococcus *framework spp. with *Alu*I**. Click here for file

Additional file 14 Dendrogram based on restriction digestion of 12 *Streptococcus *framework spp. with *Bfa*I. Click here for file

Additional file 15 **Dendrogram based on restriction digestion of 12 *Streptococcus *framework spp. with *Hae*III**. Click here for file

Additional file 16 **Dendrogram based on restriction digestion of 12 *Streptococcus *framework spp. with *Msp*I**. Click here for file

Additional file 17 **Dendrogram based on restriction digestion of 12 *Streptococcus *framework spp. with *Rsa*I**. Click here for file

Additional file 18 **Dendrogram based on restriction digestion of 12 *Streptococcus *framework spp. with *Sau*3AI**. Click here for file
