# Supplementary figures and images for: Exploring internal features of 16S rRNA gene for identification of clinically relevant species of the genus Streptococcus
Source: Ann Clin Microbiol Antimicrob. 2011 Jun 25;10:28. doi: 10.1186/1476-0711-10-28 (PMC3151204; doi:10.1186/1476-0711-10-28)

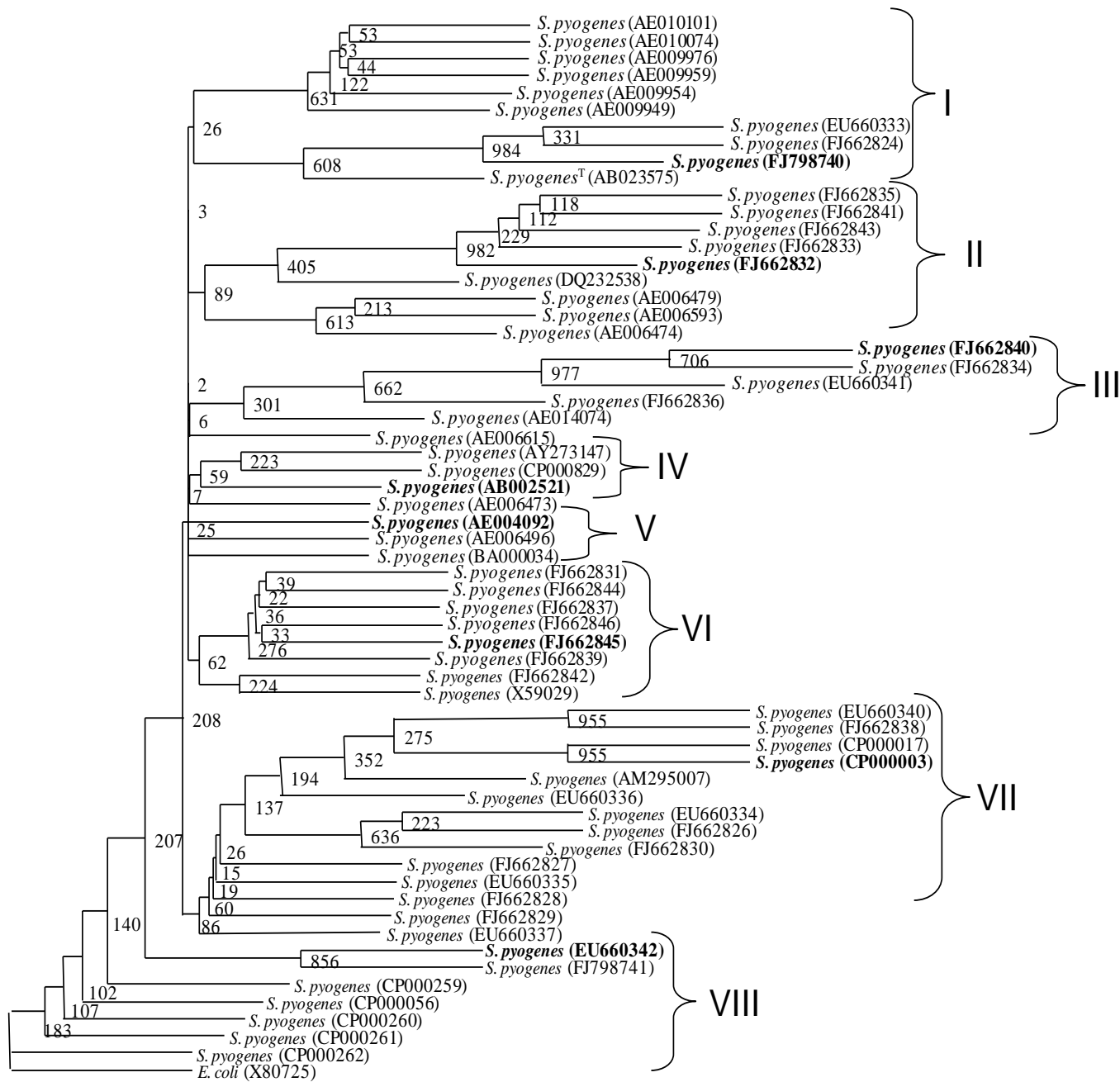

Supplement: Additional file 1 — Phylogenetic tree based on 61, 16S rRNA gene sequences of Streptococcus pyogenes. The tree was constructed by neighbour-joining method with Jukes and Cantor correction. The numbers at node represent bootstrap values (based on 1000 resampling). The accession numbers are shown in parenthesis. Bold sequences indicate those which are used for final framework construction. [file 1476-0711-10-28-S1.PDF]

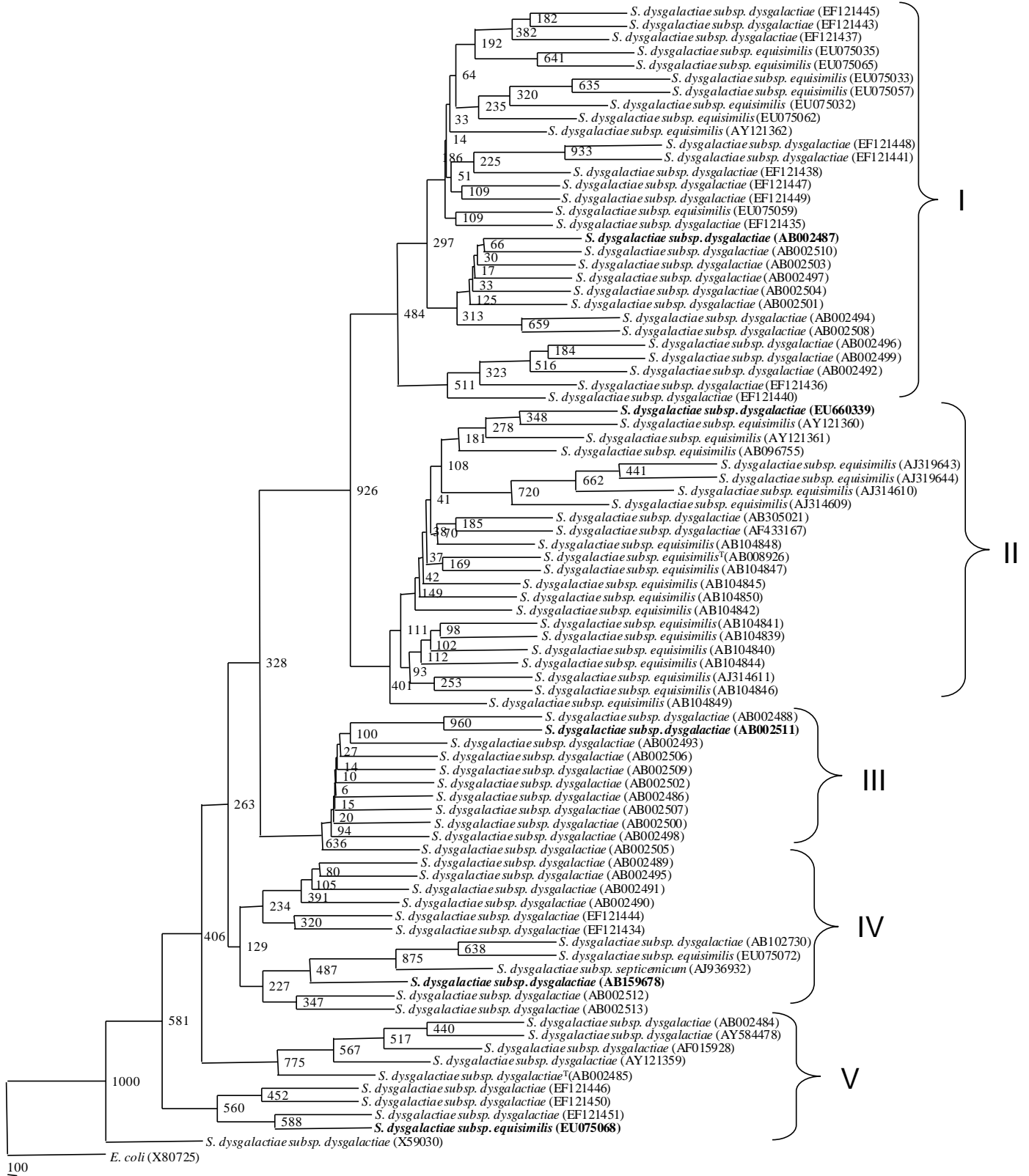

Supplement: Additional file 2 — Phylogenetic tree based on 86, 16S rRNA gene sequences of Streptococcus dysgalactiae. The tree was constructed by neighbour-joining method with Jukes and Cantor correction. The numbers at node represent bootstrap values (based on 1000 resampling). The accession numbers are shown in parenthesis. Bold sequences indicate those which are used for final framework construction. [file 1476-0711-10-28-S2.PDF]

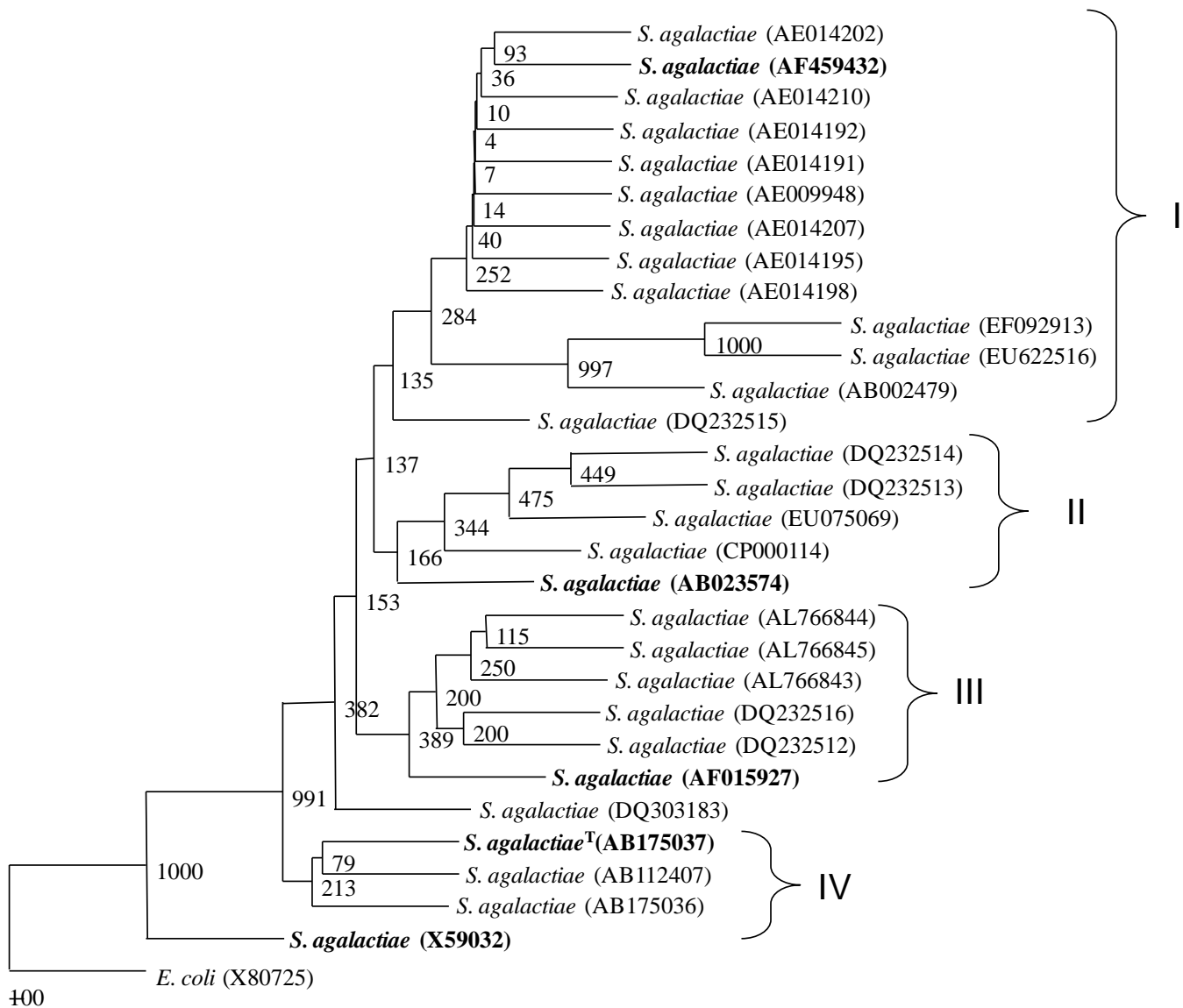

Supplement: Additional file 3 — Phylogenetic tree based on 29, 16S rRNA gene sequences of Streptococcus agalactiae. The tree was constructed by neighbour-joining method with Jukes and Cantor correction. The numbers at node represent bootstrap values (based on 1000 resampling). The accession numbers are shown in parenthesis. Bold sequences indicate those which are used for final framework construction. [file 1476-0711-10-28-S3.PDF]

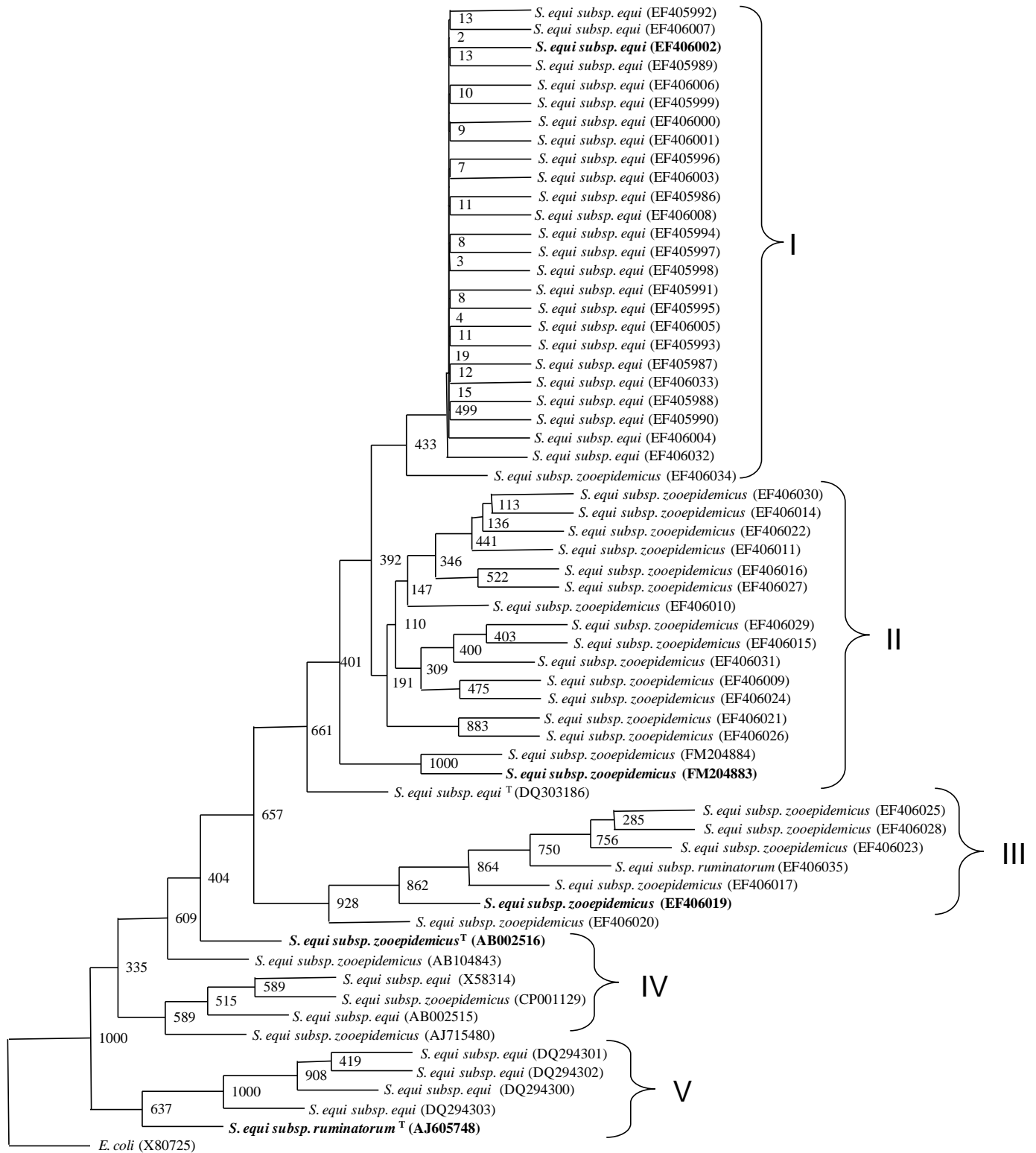

Supplement: Additional file 4 — Phylogenetic tree based on 61, 16S rRNA gene sequences of Streptococcus equi. The tree was constructed by neighbour-joining method with Jukes and Cantor correction. The numbers at node represent bootstrap values (based on 1000 resampling). The accession numbers are shown in parenthesis. Bold sequences indicate those which are used for final framework construction. [file 1476-0711-10-28-S4.PDF]

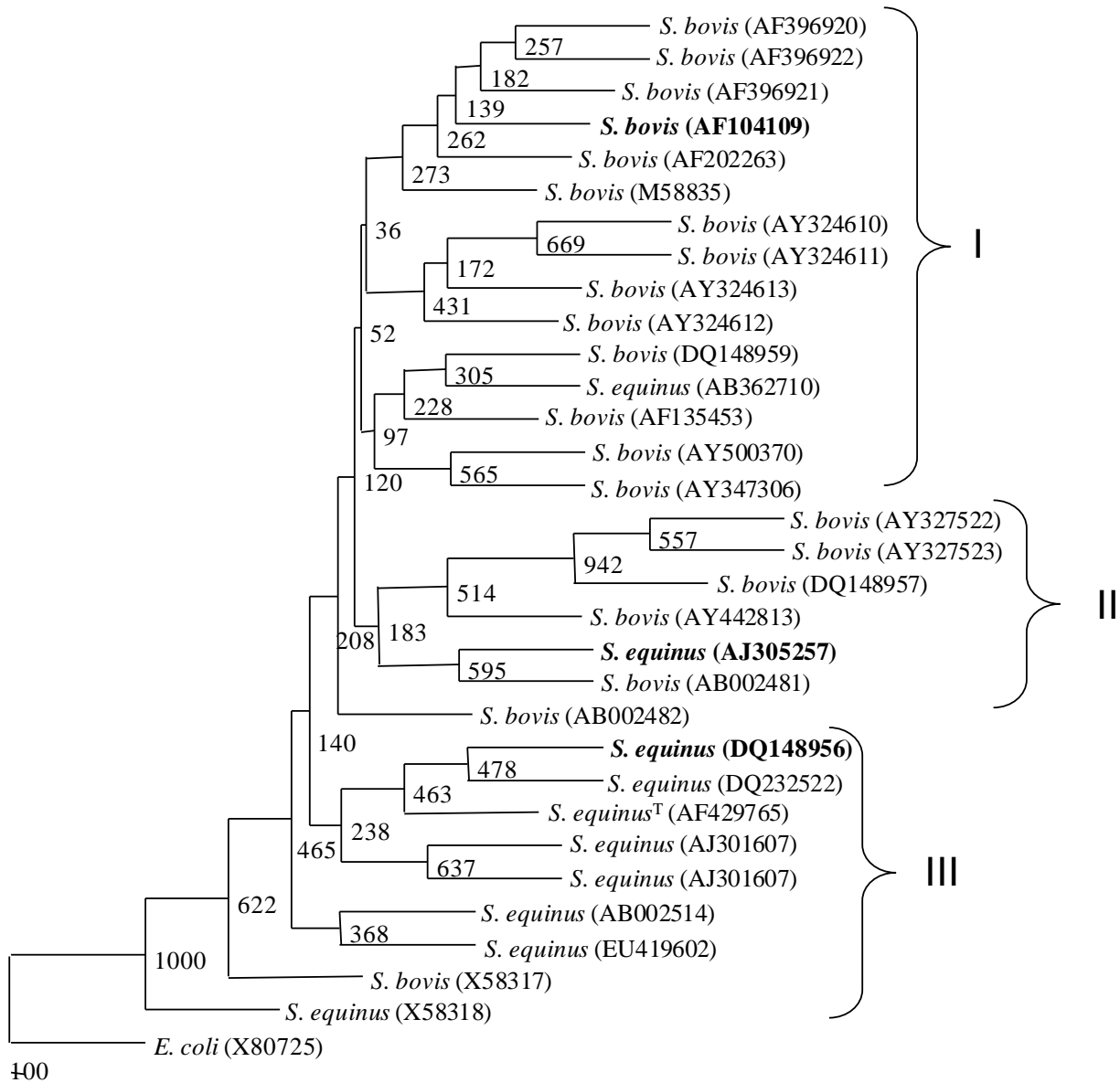

Supplement: Additional file 5 — Phylogenetic tree based on 31, 16S rRNA gene sequences of Streptococcus bovis-equinus. The tree was constructed by neighbour-joining method with Jukes and Cantor correction. The numbers at node represent bootstrap values (based on 1000 resampling). The accession numbers are shown in parenthesis. Bold sequences indicate those which are used for final framework construction. [file 1476-0711-10-28-S5.PDF]

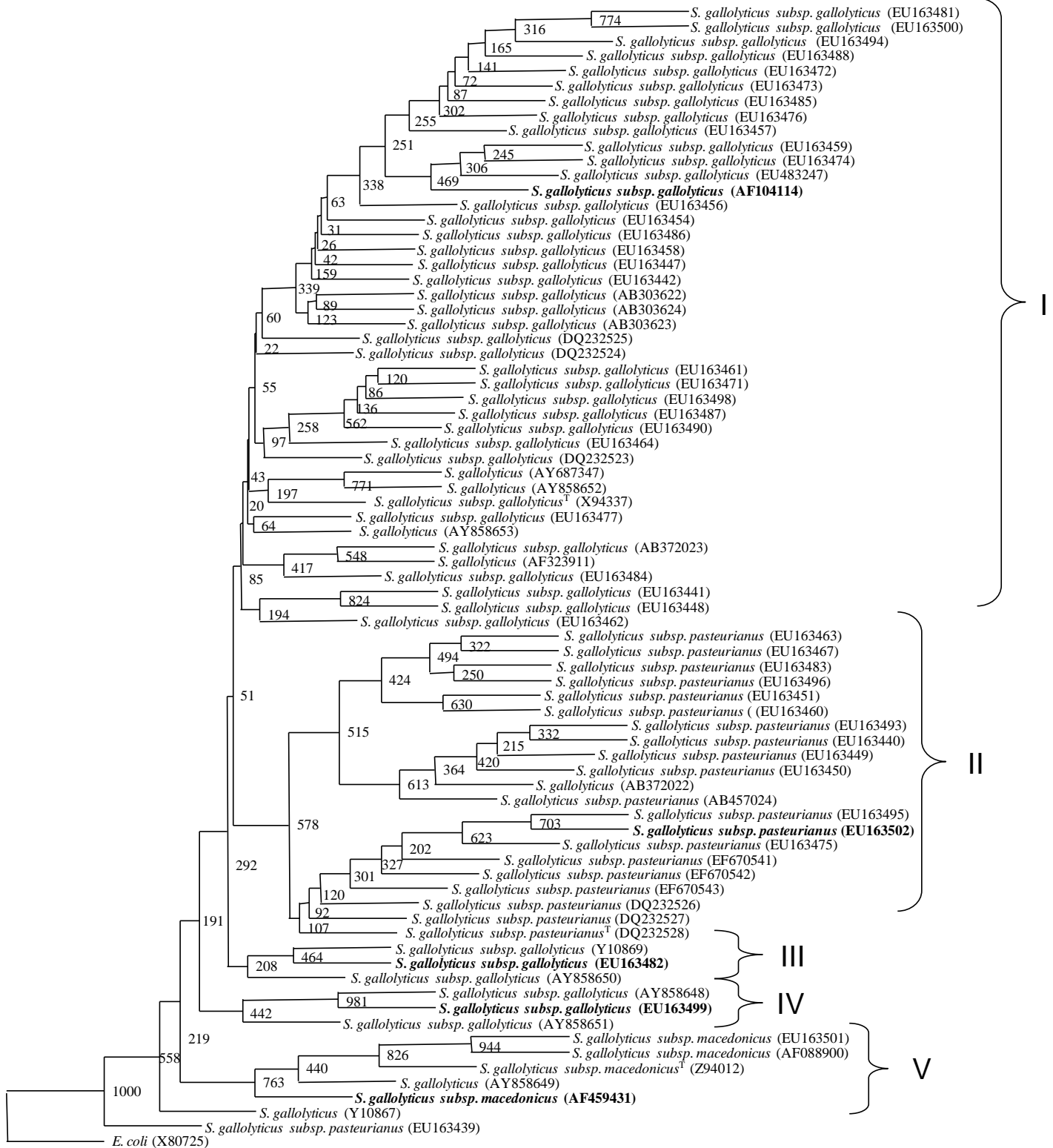

Supplement: Additional file 6 — Phylogenetic tree based on 76, 16S rRNA gene sequences of Streptococcus gallolyticus. The tree was constructed by neighbour-joining method with Jukes and Cantor correction. The numbers at node represent bootstrap values (based on 1000 resampling). The accession numbers are shown in parenthesis. Bold sequences indicate those which are used for final framework construction. [file 1476-0711-10-28-S6.PDF]

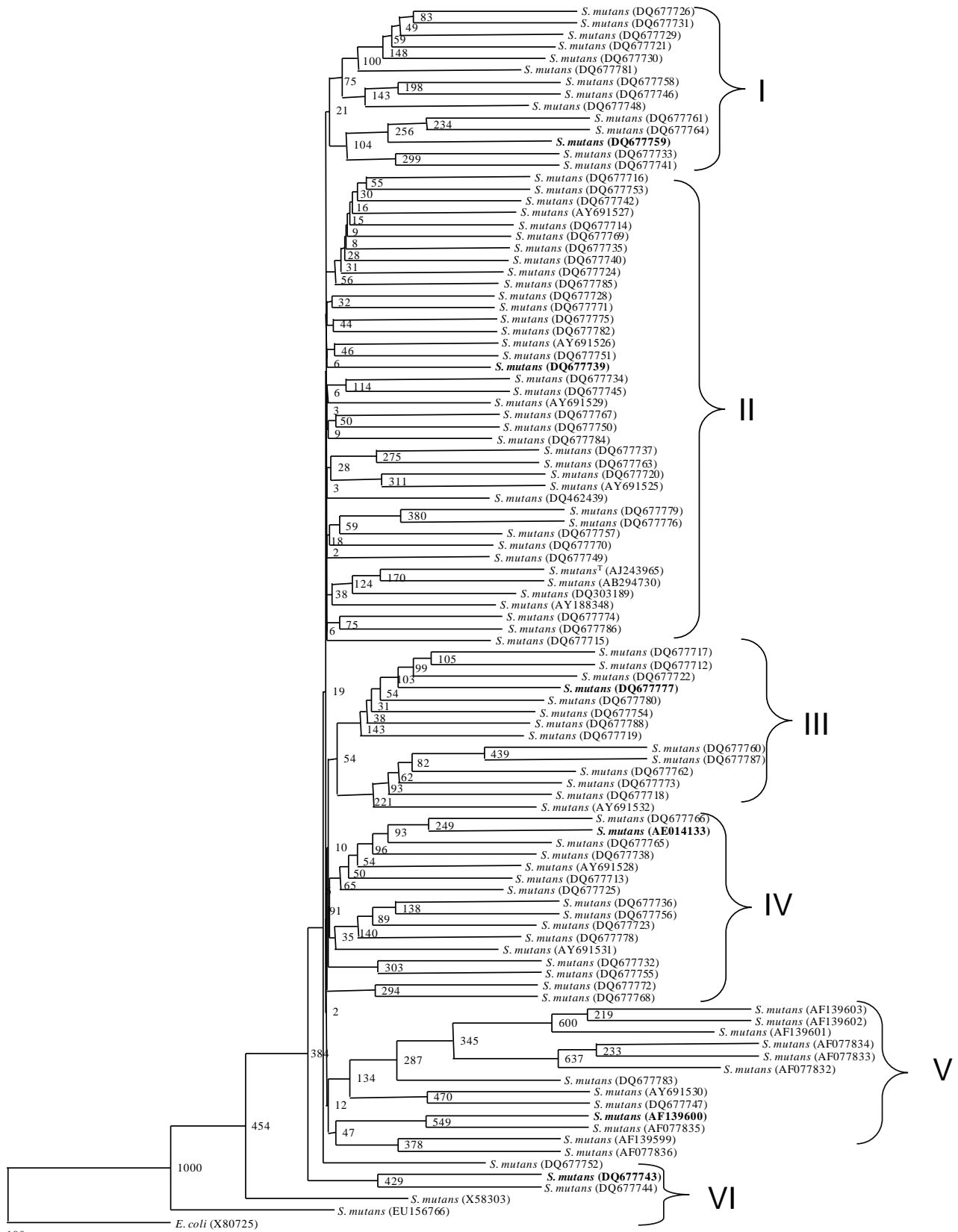

Supplement: Additional file 7 — Phylogenetic tree based on 102, 16S rRNA gene sequences of Streptococcus mutans. The tree was constructed by neighbour-joining method with Jukes and Cantor correction. The numbers at node represent bootstrap values (based on 1000 resampling). The accession numbers are shown in parenthesis. Bold sequences indicate those which are used for final framework construction. [file 1476-0711-10-28-S7.PDF]

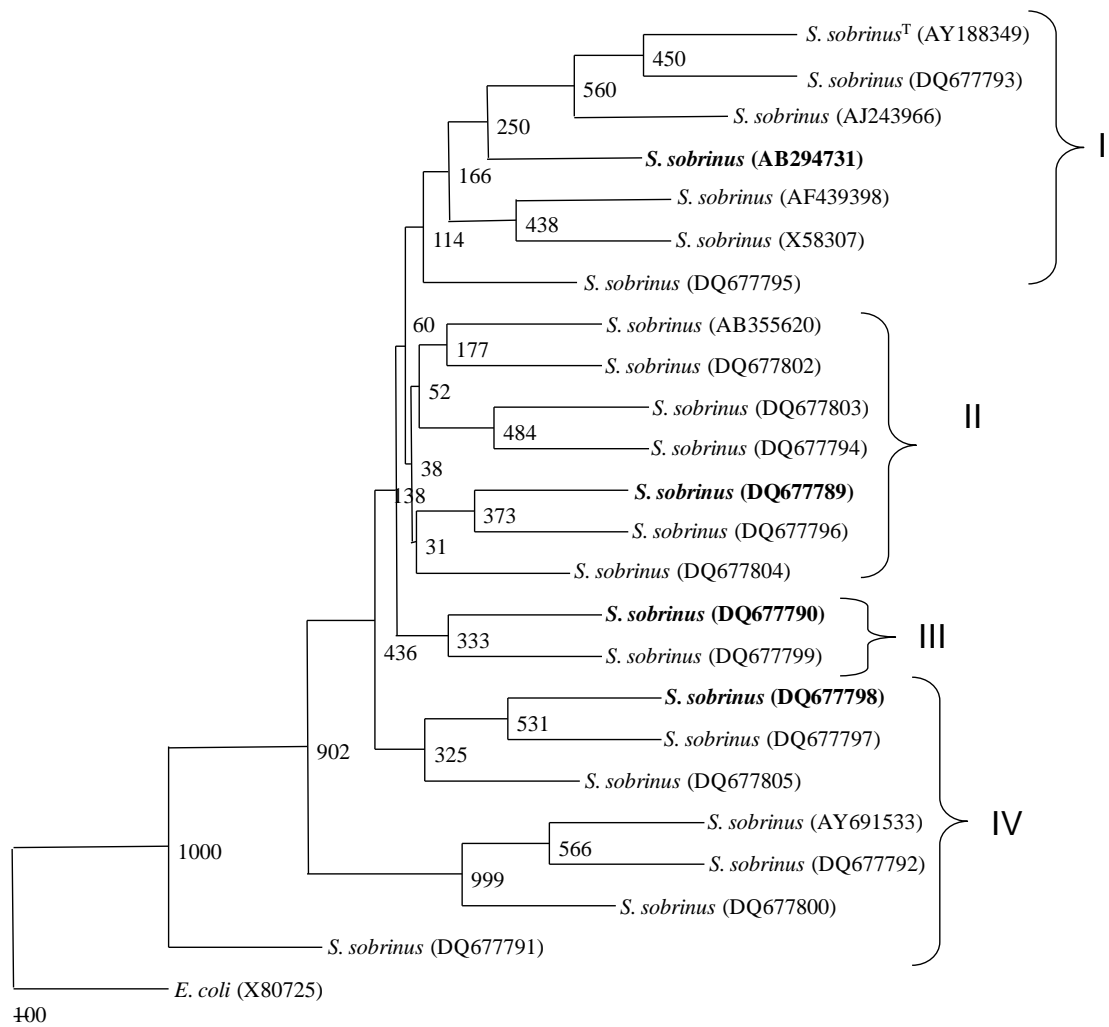

Supplement: Additional file 8 — Phylogenetic tree based on 23, 16S rRNA gene sequences of Streptococcus sobrinus. The tree was constructed by neighbour-joining method with Jukes and Cantor correction. The numbers at node represent bootstrap values (based on 1000 resampling). The accession numbers are shown in parenthesis. Bold sequences indicate those which are used for final framework construction. [file 1476-0711-10-28-S8.PDF]

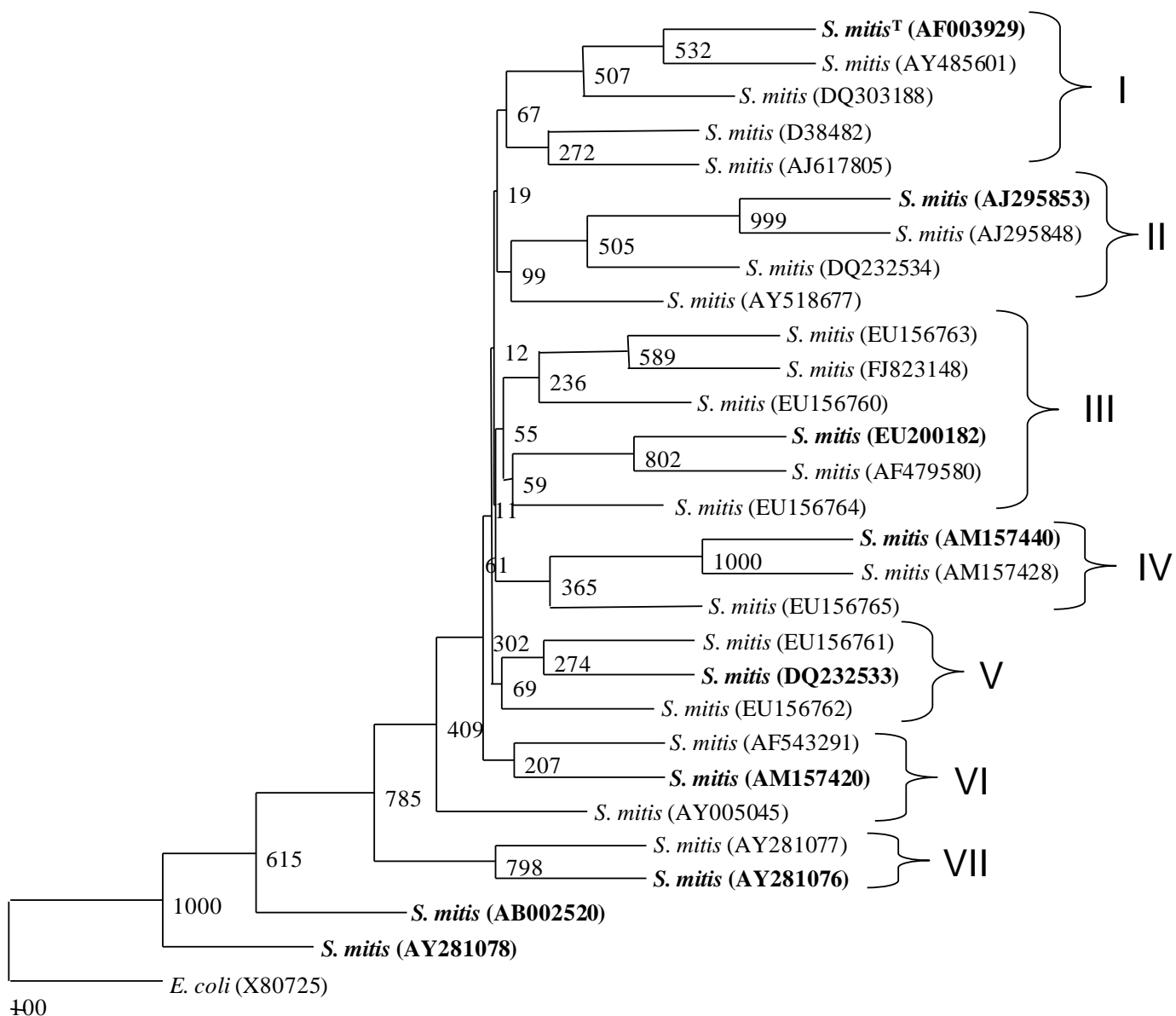

Supplement: Additional file 9 — Phylogenetic tree based on 28, 16S rRNA gene sequences of Streptococcus mitis. The tree was constructed by neighbour-joining method with Jukes and Cantor correction. The numbers at node represent bootstrap values (based on 1000 resampling). The accession numbers are shown in parenthesis. Bold sequences indicate those which are used for final framework construction. [file 1476-0711-10-28-S9.PDF]

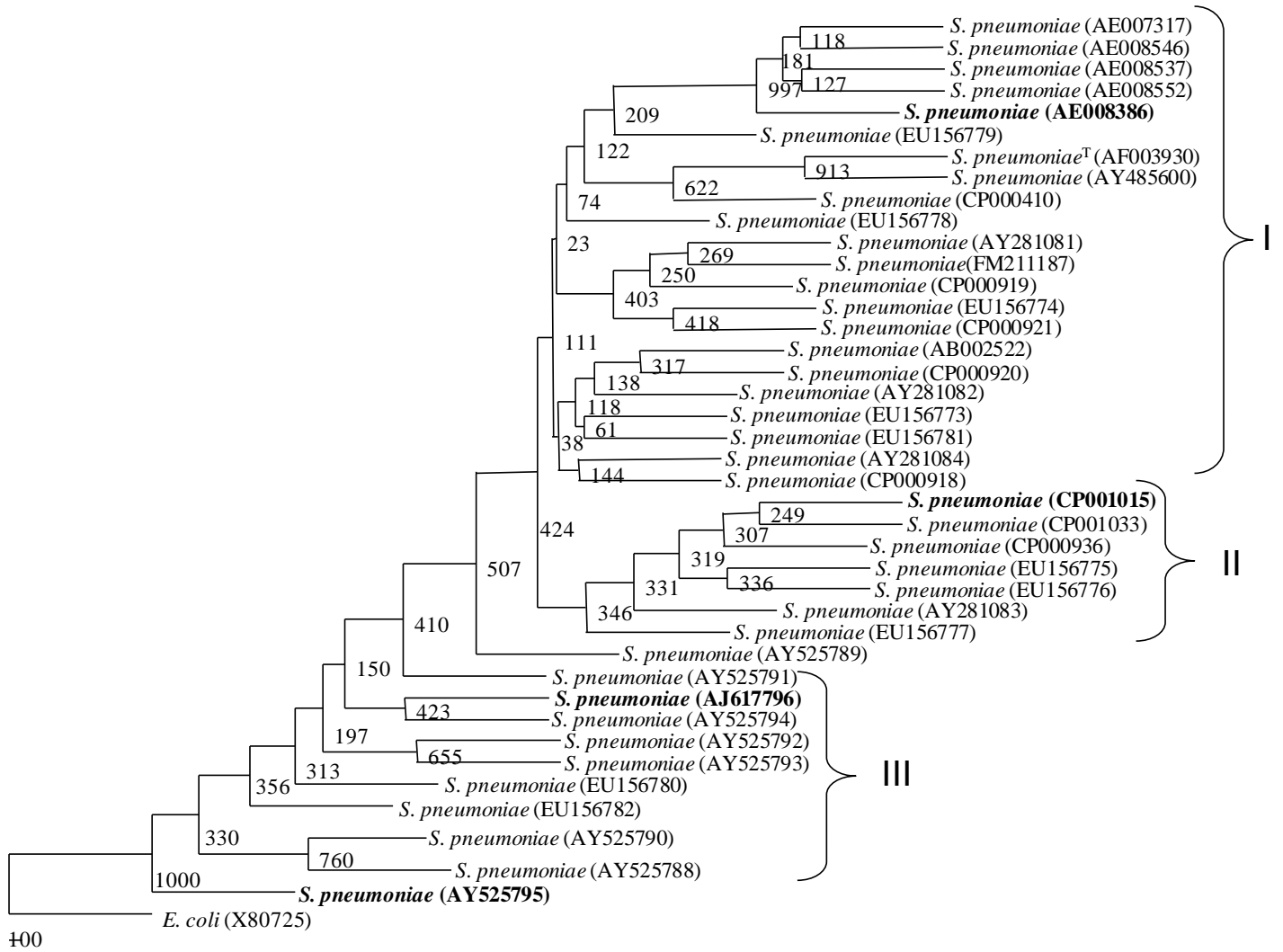

Supplement: Additional file 10 — Phylogenetic tree based on 41, 16S rRNA gene sequences of Streptococcus pneumoniae. The tree was constructed by neighbour-joining method with Jukes and Cantor correction. The numbers at node represent bootstrap values (based on 1000 resampling). The accession numbers are shown in parenthesis. Bold sequences indicate those which are used for final framework construction. [file 1476-0711-10-28-S10.PDF]

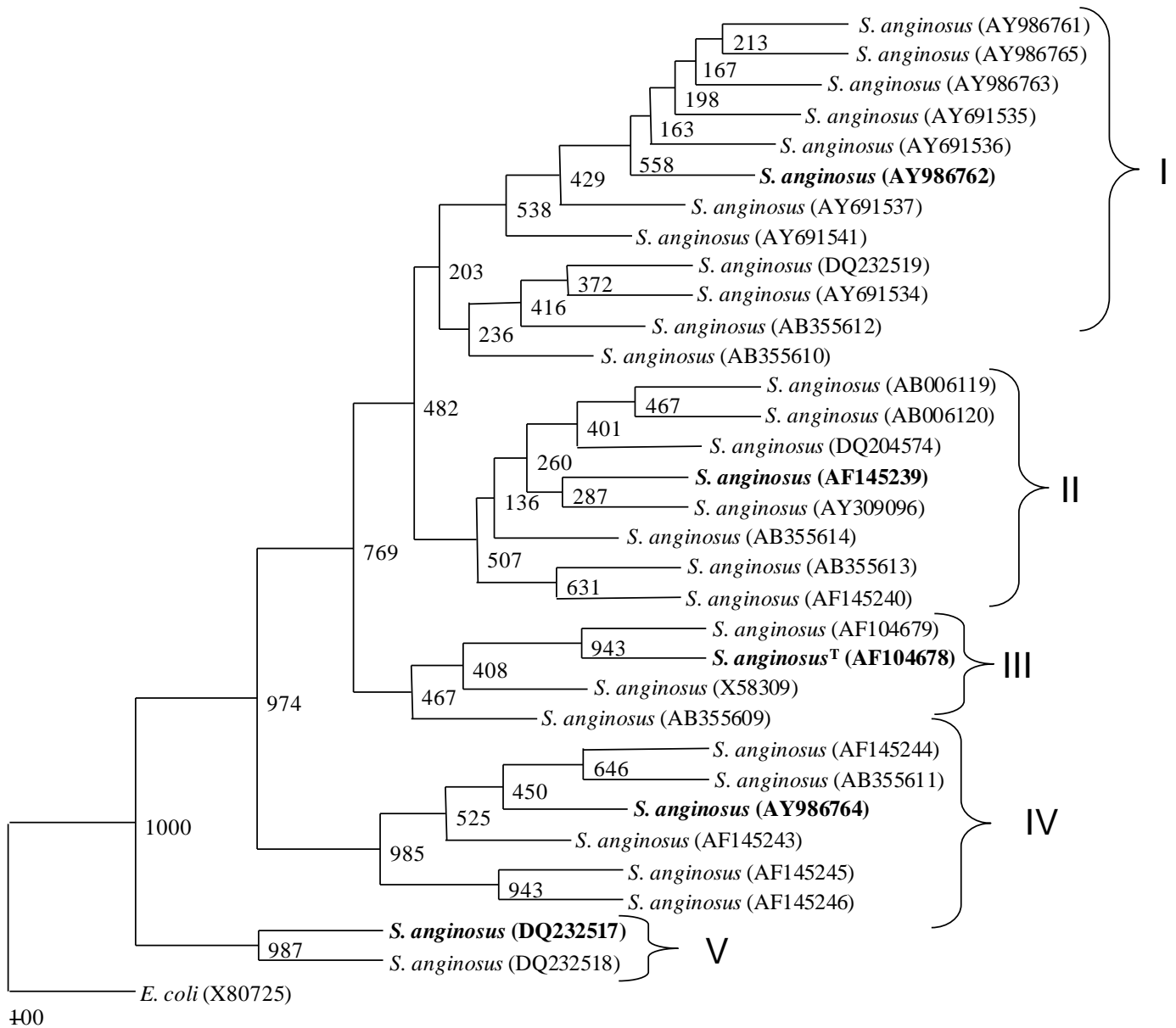

Supplement: Additional file 11 — Phylogenetic tree based on 32, 16S rRNA gene sequences of Streptococcus anginosus. The tree was constructed by neighbour-joining method with Jukes and Cantor correction. The numbers at node represent bootstrap values (based on 1000 resampling). The accession numbers are shown in parenthesis. Bold sequences indicate those which are used for final framework construction. [file 1476-0711-10-28-S11.PDF]

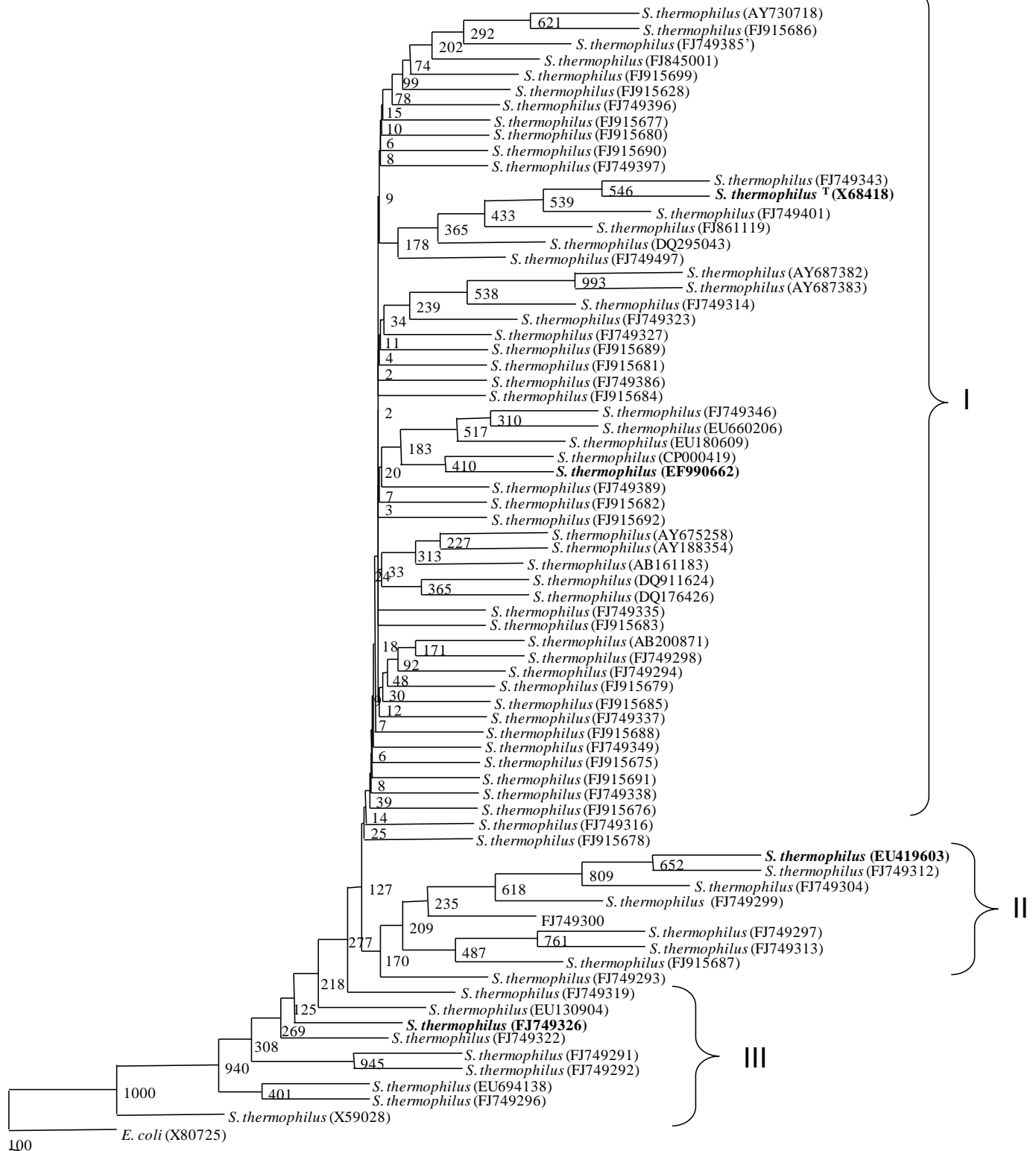

Supplement: Additional file 12 — Phylogenetic tree based on 73, 16S rRNA gene sequences of Streptococcus thermophilus. The tree was constructed by neighbour-joining method with Jukes and Cantor correction. The numbers at node represent bootstrap values (based on 1000 resampling). The accession numbers are shown in parenthesis. Bold sequences indicate those which are used for final framework construction. [file 1476-0711-10-28-S12.PDF]

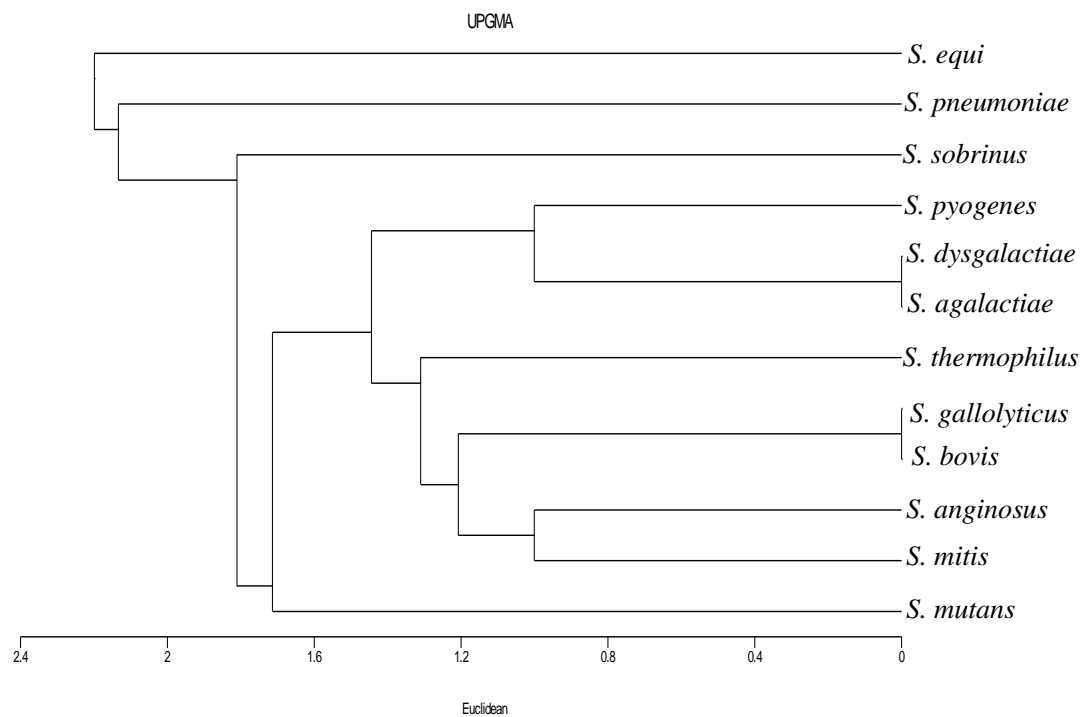

**Supplementary Fig. S13**

Supplement: Additional file 13 — Dendrogram based on restriction digestion of 12 Streptococcus framework spp. with AluI. [file 1476-0711-10-28-S13.PDF]

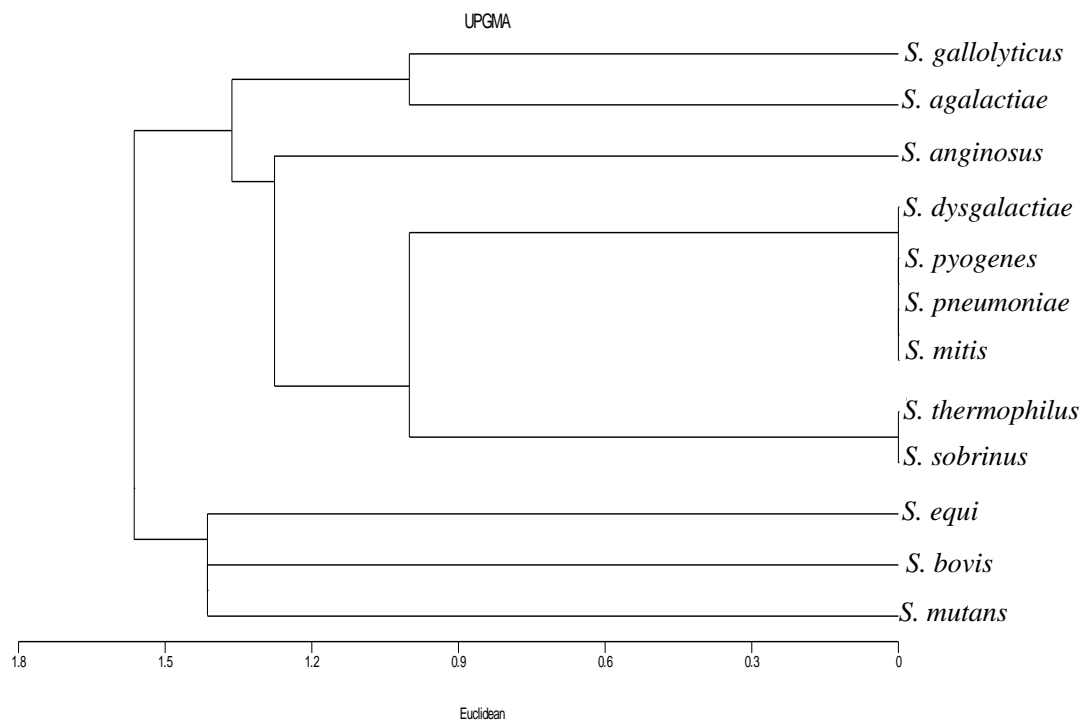

**Supplementary Fig. S14**

Supplement: Additional file 14 — Dendrogram based on restriction digestion of 12 Streptococcus framework spp. with BfaI. [file 1476-0711-10-28-S14.PDF]

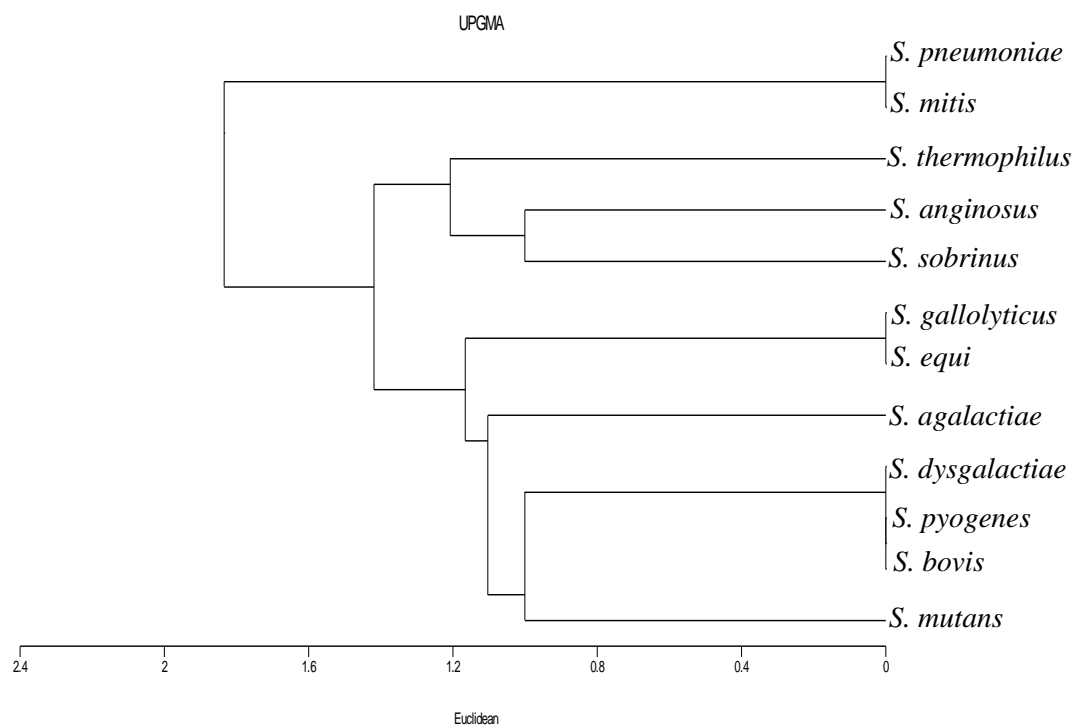

**Supplementary Fig. S15**

Supplement: Additional file 15 — Dendrogram based on restriction digestion of 12 Streptococcus framework spp. with HaeIII. [file 1476-0711-10-28-S15.PDF]

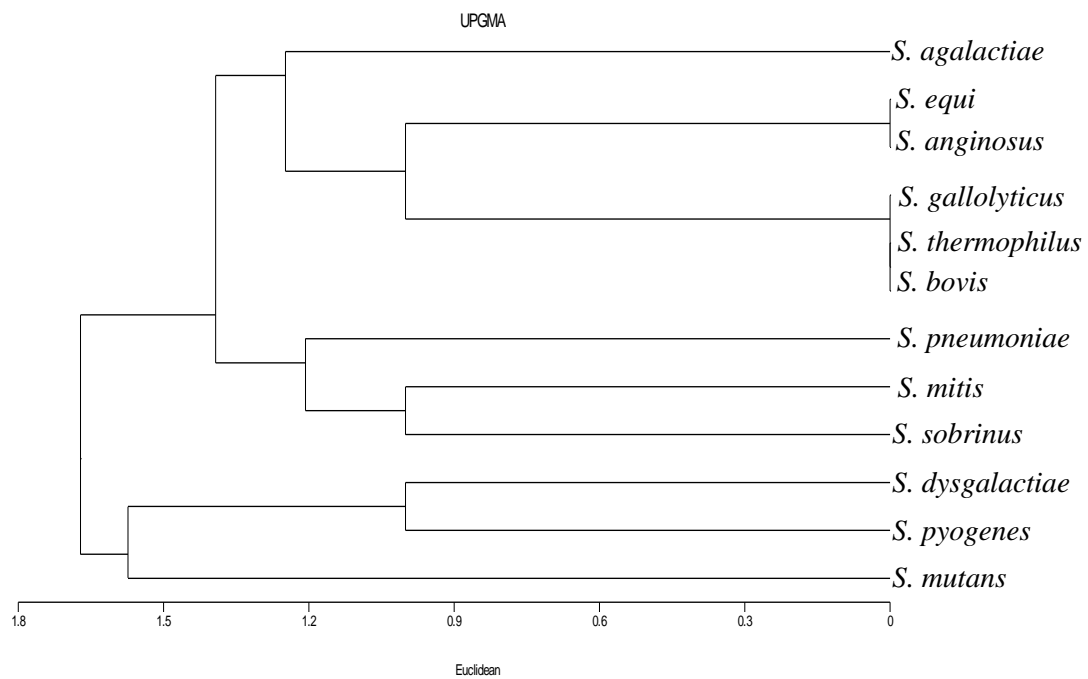

**Supplementary Fig. S16**

Supplement: Additional file 16 — Dendrogram based on restriction digestion of 12 Streptococcus framework spp. with MspI. [file 1476-0711-10-28-S16.PDF]

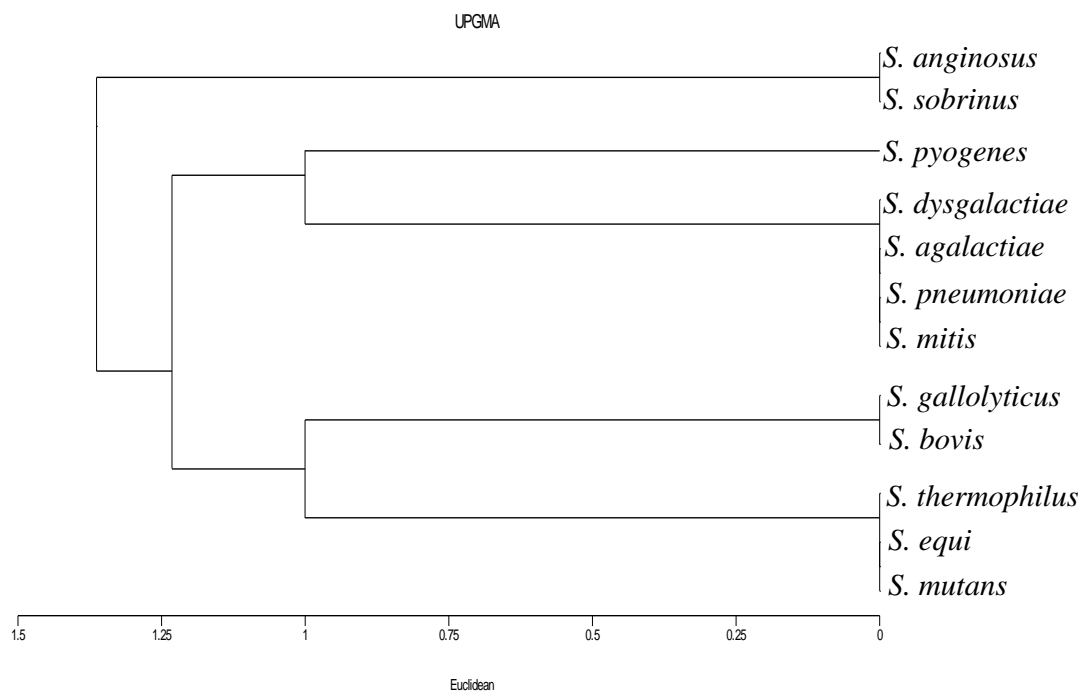

**Supplementary Fig. S17**

Supplement: Additional file 17 — Dendrogram based on restriction digestion of 12 Streptococcus framework spp. with RsaI. [file 1476-0711-10-28-S17.PDF]

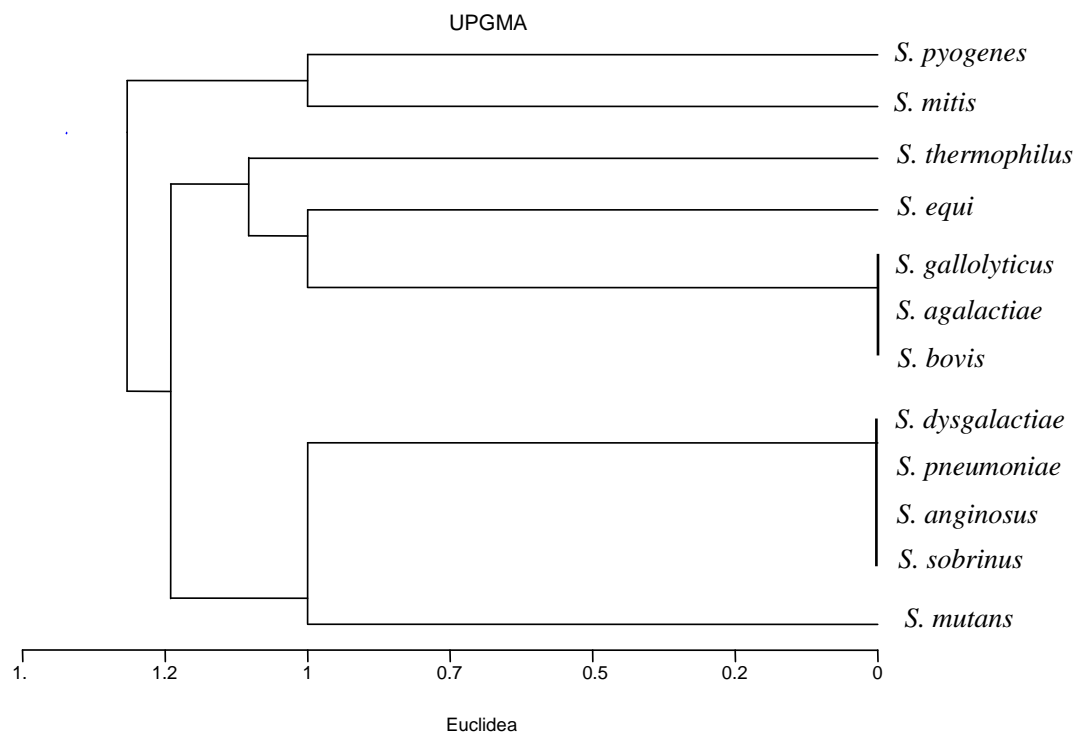

**Supplementary Fig. S18**

Supplement: Additional file 18 — Dendrogram based on restriction digestion of 12 Streptococcus framework spp. with Sau3AI. [file 1476-0711-10-28-S18.PDF]
